# Comparing DNA isolation methods for forest trees: quality, plastic footprint, and time-efficiency

**DOI:** 10.1186/s13007-023-01086-y

**Published:** 2023-10-19

**Authors:** Laura Guillardín, John J. MacKay

**Affiliations:** Department of Biology, University of Oxford, South Parks Road, Oxford, OX1 3RB United Kingdom

**Keywords:** DNA isolation, DNA quality, Plastic footprint, Time-efficient, Sustainability, Forest Trees, DNA Barcoding

## Abstract

**Background:**

Genetic and genomic studies are seeing an increase in sample sizes together with a wider range of species investigated in response to environmental change concerns. In turn, these changes may come with challenges including the time and difficulty to isolate nucleic acids (DNA or RNA), the sequencing cost and environmental impacts of the growing amount of plastic waste generated in the process. *Pseudotsuga menziesii* var. *menziesii* (Mirbel) Franco (PM), *Tsuga heterophylla* (Raf.) Sarg. (TH) and *Thuja plicata* Donn ex D.Don (TP) are conifer species found in diverse woodlands both as natives and naturalized exotics. Our study was carried out whilst investigating their genetics to understand their population structure and potential for adaptation.

**Results:**

In the present study, we compared two different DNA isolation methods, i.e., spin-column DNeasy plant mini kit (QIAGEN), and temperature-driven enzymatic cocktail Plant DNA Extraction (MicroGEM). The quantity of recovered DNA and the quality of DNA were assessed along with the plastic footprint and time needed for three tree species. Both methods were optimised and proven to provide enough DNA for each studied species. The yield of DNA for each method depended on the species: QIAGEN showed higher yield in *P. menziesii* and *T. heterophylla*, while *T*. *plicata* recovered similar amount of DNA for both methods. The DNA quality was investigated using DNA barcoding techniques by confirming species identity and species discrimination. No difference was detected in the PCR amplification of the two barcoding loci, (*rbcL* and *trnH-psbA*), and the recovered sequences between DNA isolation methods. Measurement of the plastic use and the processing time per sample indicated that MicroGEM had a 52.64% lower plastic footprint and was 51.8% faster than QIAGEN.

**Conclusions:**

QIAGEN gave higher yields in two of the species although both methods showed similar quality results across all species. However, MicroGEM was clearly advantageous to decrease the plastic footprint and improve the time efficiency. Overall, MicroGEM recovers sufficient and reliable DNA to perform common downstream analyses such as PCR and sequencing. Our findings illustrate the benefits of research and efforts towards developing more sustainable methods and techniques to reduce the environmental footprint of molecular analyses.

## Background

Genetic and genomic analyses often benefit from a large sample set to reliably detect associations and identify patterns relevant to key biological questions [1] due to higher statistical power and accuracy [2]. However, larger sample sizes require more resources, such as time to collect and process the samples and the volume of consumables used in the laboratory [3]. Analyses commonly used include Genome-wide association studies (GWAS) [4], genomic selection (GS) and Genome environment association (GEA) to scan genomes for associations with complex traits of interest as in tree breeding programs [5] where the power to detect loci with small effects may be constrained by the sample size [6]. The minimum sample size for each genetic analysis varies depending on the research question, the genetic markers used, the biological system and the available resources. Environmental change [7] and its effects on natural habitats as well as in the distribution, population size and genetic diversity [7–11] increases the number of species at risk and in need to be studied [10]. Therefore, both the range and the size of studies involving molecular analyses are increasing [12].

Molecular studies on non-model tree species may face challenges including obtaining high-quality DNA and RNA, accessing wild populations, the complexity and size of their genome, and the limited available genomic resources [13]. These factors increase the complexity and the resource intensity of genomic studies in trees compared to studies of many other organisms [14]. The challenges of obtaining high-quality DNA and RNA from trees include the difficulty of breaking down the cell walls to release the nucleic acids [15] and the inhibitory effects of secondary metabolites and polysaccharides on downstream applications such as PCR (polymerase chain reaction), sequencing and genotyping. Simple and robust protocols for isolation of high-quality nucleic acids from tree tissues that are suitable for downstream applications would help to overcome these challenges.

The main limitations when designing genomic experiments with large sample sizes include the time and difficulty to isolate nucleic acids (DNA or RNA), the sequencing cost and the environmental impact of plastic waste generated in the process. Sequencing platforms have reduced nucleic acid sequencing costs over the last two decades [16], making sequencing more accessible to researchers studying non-model species and feasible to study larger sample sizes. Some cost-effective pipelines, workflows and strategies have also been developed to reduce costs and process time by reusing consumables [17]. Single-use plastic consumables are a significant source of waste in many scientific fields [18, 19], with an estimated use of 5.5 million tonnes per year worldwide [20]; therefore, although they are essential to perform genomic analyses, they have a large environmental impact [21]. Improved laboratory sustainability is encouraged by organizations and labels such as My Green Lab [22] and The Sustainable Laboratory Practices Working Group (SLPWG) [23] which promote green practices in the laboratory to reduce their plastic footprint. A different approach is to reuse specific plastic items, for example, Grenova Solutions [24] developed an on-bench Pipette Tip Cleaning Machine (TipNovus) that is easy to integrate into laboratory routines. Another solution is to choose or develop protocols that use less plastic without compromising the experiment's outcome [3, 25].

Several methods have been proposed to overcome the difficulties when isolating DNA from tree species. Broadly, there are five main types of DNA isolation systems including organic extraction methods which use organic solvents like phenol and chloroform [26, 27], solid-phase extraction methods that use solid matrices, such as silica to bind and purify the DNA [28], precipitation methods which use salts and ethanol to precipitate the DNA [29], enzymatic digestion methods that use individual or a combination of enzymes to digest the samples and release the DNA [30] and use of magnetic bead-based coated with an agent which binds with DNA and isolates it from the cellular suspension [31], or magnetic ionic liquids [32]. These methods are usually combined and modified depending on the experimenter's needs [17, 33].

In this study, we compared the performance of two different DNA isolation methods in three conifer forest tree species: *Pseudotsuga menziesii* var. *menziesii* (Mirbel) Franco (PM), *Tsuga heterophylla* (Raf.) Sarg. (TH) and *Thuja plicata* Donn ex D.Don (TP). They are all large evergreen coniferous trees native to western North America where they appear together in both mixed natural forests and plantations. Coniferous species, including the study species, are widely found in Europe and North America in woodlands which provide a variety of ecosystem services including timber production, carbon sequestration, biodiversity and habitat conservation, water regulation, recreation, and tourism [34, 35]. Additionally, conifer trees are commonly used as pioneer species in reforestation projects in poorer sites due to their resilience to extreme environmental conditions [36]. At the same time, conifer species are affected by the change in the climate [12] and its subsequent increasing disturbances such as the increase of forest wildfires and their intensity, longer and higher number of drought periods and pests and disease outbreaks. Therefore, conifers are largely studied organisms to shed light on the genomic mechanisms of adaptation, the association to different environments, the levels of population genetic diversity of different woodlands and the genetic resistance to pests & diseases, among others [12].

We tested the recovered DNA quantity, quality, plastic footprint and time of the following methods: (i) Plant DNA Extraction (MicroGEM International PLC, 2019) (MicroGEM) which uses a temperature-controlled enzymatic cocktail and (ii) DNeasy plant mini kit (Qiagen USA, Valencia, CA) (QIAGEN), a silica column-based method widely used to isolate DNA from conifer trees. In this context, the aim of this study is to find an efficient DNA isolation method for forest trees by looking at the recovered DNA quality, the plastic waste generated, the energy required to produce it and the total time needed to process the samples using both DNA isolation methods. We verified the quality of the DNA recovered by use of PCR with gene-specific primers followed by DNA sequencing.

## Results

### Improvement of DNA isolation methods and comparison of species

**Fig. 1:**
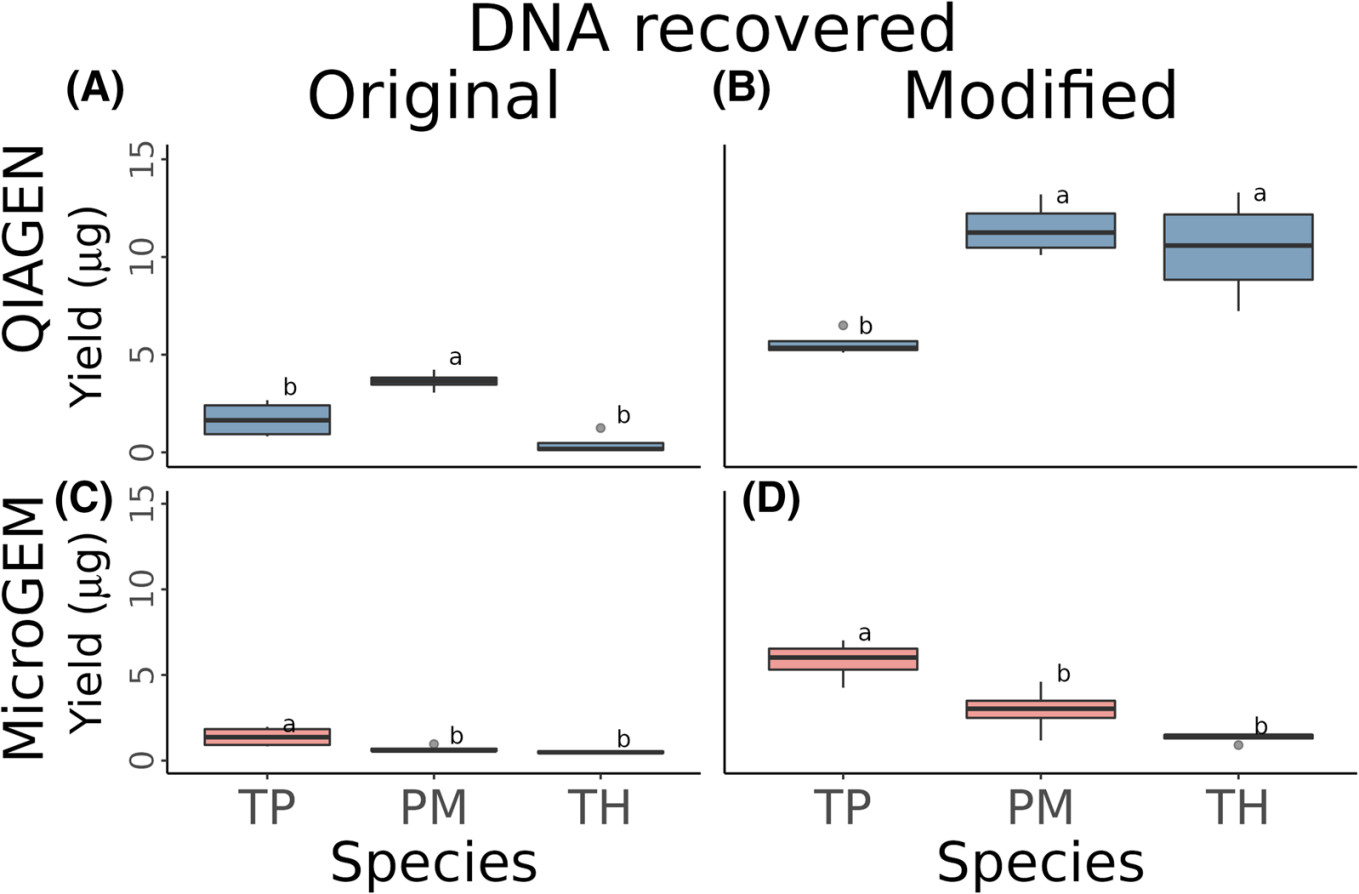
DNA yield recovered by **A** using QIAGEN original protocol, **B** QIAGEN modified protocol, **C** MicroGEM original protocol and **D** modified MicroGEM protocol. TP: *T. plicata*, PM: *P. menziesii* and TH: *T. heterophylla*. Letters represent significant differences between species for each of the protocols tested

We isolated the DNA from four samples in each of the three study species by using MicroGEM and QIAGEN original and improved protocols (see methods for details). The improvements to DNA isolation methods increased the DNA recovered both for the MicroGEM and QIAGEN methods in *T. plicata*, *P. menziesii *and *T. heterophylla* (Fig. 1). The data suggest that DNA yields were generally higher with QIAGEN, except for *T. plicata* where the results were similar across methods (Fig. 1B, D). However, statistical comparison of modified methods and species showed significant effects of species and the interaction of species and methods but not between methods (Table 1). A post hoc Tukey test among species within methods showed that *P. menziesii* yielded significantly better than the other species when using the original QIAGEN protocol, while *T. plicata* showed significantly better results when using the original MicroGEM method (Fig. 1A, C). The modified QIAGEN showed *P. menziesii* and *T. heterophylla* grouping together and yielding significantly higher than *T. plicata* results while with MicroGEM modified protocol *T. plicata* showed significantly higher yields compared to the other species (Fig. 1B, D).

**Table 1:** Anova table of factors affecting DNA yield recovered including methods, species and their interaction

Anova table (Type III tests)	Sum Sq	Df	F value	Pr (> F)	Significance
(Intercept)	124434025.00	1	57.63	5.13E-07	***
Method	126253.13	1	0.06	8.12E-01	
Species	78713516.67	2	18.23	4.71E-05	***
Method:Species	109300794.75	2	25.31	5.88E-06	***
Residuals	38865031.50	18			

*Sum Sq* Sum of squares, *Df* Degrees of freedom, *Pr* Probability

'***' corresponds to a P. value of 0.001

Overall, the modified procedures yielded more than 1 µg across all the species starting from a minimum of 10 mg when using MicroGEM and 20 mg with QIAGEN of dried tissue (except for TH4 extracted with MicroGEM, Table 2), which is sufficient for many downstream analyses. Yields above 10 µg were obtained in *P. menziesii* and most of *T. heterophylla* but only for QIAGEN method (Table 2). The samples extracted with the modified protocols of both DNA isolation methods will be used for the subsequent analyses.

**Table 2:** DNA yield across species with improved methods

Sample ID	Conc.(ng/µl) MicroGEM	Yield (µg) MicroGEM	Conc.(ng/µl) QIAGEN	Yield (µg) QIAGEN
TH1	17.8	1.42	72.3	7.23
TH2	21.2	1.48	133.0	13.30
TH3	19.3	1.54	118.0	11.80
TH4	15.1	0.91	93.7	9.37
TP1	78.0	7.02	51.1	5.11
TP2	75.0	6.38	65.0	6.50
TP3	53.2	4.26	52.8	5.28
TP4	70.8	5.66	54.2	5.42
PM1	65.8	4.61	119.0	11.90
PM2	41.9	2.93	106.0	10.60
PM3	16.8	1.18	132.0	13.20
PM4	44.6	3.12	101.0	10.10

### DNA recovered quality

**Fig. 2:**
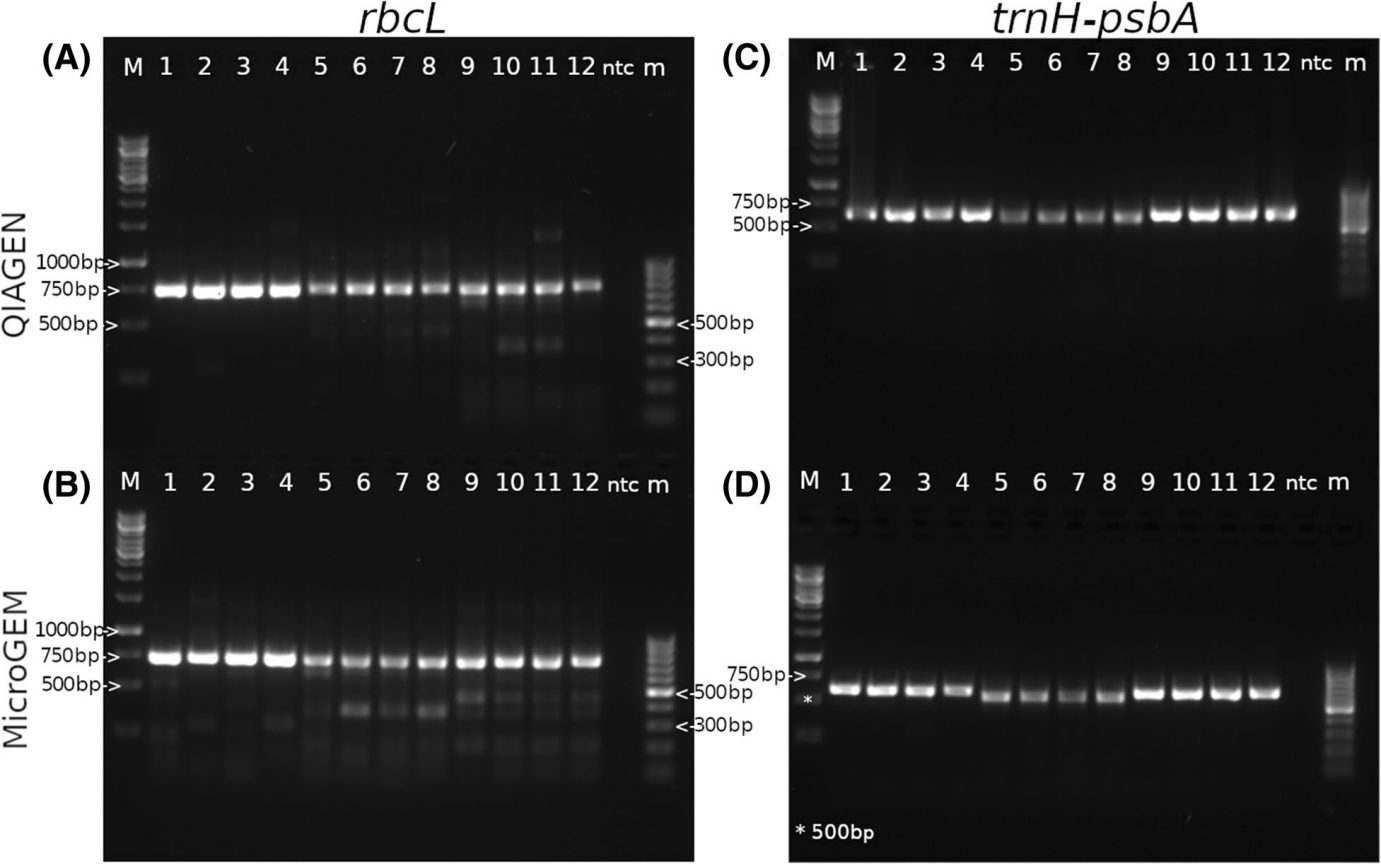
*rbcL* PCR product for **A** QIAGEN **B** MicroGEM samples and *trnH-psbA* for **C** QIAGEN and **D** MicroGEM samples. Sample names: M: 1 Kb molecular-weight size marker, 1:TH1, 2:TH2, 3:TH3, 4:TH4, 5:TP1, 6:TP2, 7:TP3, 8:TP4, 9:PM1, 10:PM2, 11:PM3, 12:PM4, ntc: non-template control, m: 100 bp molecular size marker

The quality of the DNA recovered from each method was analysed with standard DNA Barcoding techniques with commonly used gene-specific primers, the large subunit of the ribulose-bisphosphate carboxylase gene (*rbcL*) and the plastid *trnH-psbA* intergenic spacer. Major amplicons of the expected size (Table 8) were detected in all the samples tested for both barcodes *rbcL* and *trnH-psbA* (Table 3, Fig. 2). Other minor bands appeared in the *rbcL* PCR products as displayed in the gel images while none were detected in the *trnH-psbA* gel (Fig. 2, Table 3). There were no differences between DNA isolation methods in *trnH-psbA* PCR products, but samples extracted with MicroGEM show minor bands in 75% of the *rbcL* products compared to only 33.3% of QIAGEN samples (Fig. 2A, C).

**Table 3:** Summary of PCR and Sequencing results for each DNA isolation method per species and barcode marker

		PCR					Sequencing		
		*rbcL*			*trnH-psbA*		*rbcL*		*trnH-psbA*
DNA isolation Method	Species	N	Major amplicon detected %	Minor bands detected %	Major amplicon detected %	Minor bands detected %	N	Expected seq. length %	Expected seq. length %
QIAGEN	TP	4	100	50	100	0	2	100	100
	PM		100	50	100	0		50	100
	TH		100	0	100	0		100	100
MicroGEM	TP		100	75	100	0		50	100
	PM		100	100	100	0		50	100
	TH		100	50	100	0		100	100

Every *trnH-psbA* sequence retrieved was of the expected length amplicon size while 25% of the *rbcL* sequences were unreliable (Table 3). When looking at the differences between the DNA isolation methods, one QIAGEN sample and two MicroGEM samples could not be sequenced satisfactorily for *rbcL*.

We submitted the *rbcL* and *trnH-psbA* recovered sequences to BOLD and GENBANK, respectively to verify the species identities of the tested samples. All sequences returned the correct species identification, except for *rbcL* in samples whose sequence length was shorter than the expected size (Table 4). Two of the problematic sequences were from samples isolated with MicroGEM (*T. plicata* and *P. menziesii*) and one from QIAGEN (*P. menziesii*) (Table 4).

**Table 4:** Species identification using GENEBANK and BOLD datasets for both barcodes

DNA isolation method	Species	N	BOLD *rbcL*	GENBANK *trnH-psbA*
QIAGEN	TP	2	2	2
	PM	2	1	2
	TH	2	2	2
MicroGEM	TP	2	1	2
	PM	2	1	2
	TH	2	2	2

*N* Number of samples

The sequences obtained of the *trnH-psbA* amplicons were clustered using USEARCH v.11 60 to assess the species discrimination efficacy of both methods. A clustering was determined for the three species and the samples were classified in species-specific clusters with a level of similarity above 99% in every case, without any difference between DNA isolation methods.

### DNA obtained on large populations

Populations with larger numbers of individuals have been processed in two of the species described above with both methods in a parallel project (Table 5). The QIAGEN method gave higher mean yields for *T. plicata* than *P. menziesii* with the latter also being more consistent as shown by lower levels of variation (CV) (Table 5). The yield recovered from *T. plicata* samples was lower and slightly more variable when using MicroGEM than QIAGEN (Table 5).

**Table 5:** Summary table of the DNA yield recovered in a larger project when using both DNA isolation methods

Method	Species	Yield Mean (µg)	Yield CV %	N
QIAGEN	TP	9.603	59.24	79
	PM	7.766	40.37	1146
MicroGEM	TP	3.312	66.09	472

*CV* Coefficient variation, *N* Number of samples

### Plastic footprint and time needed for DNA isolations

**Fig. 3:**
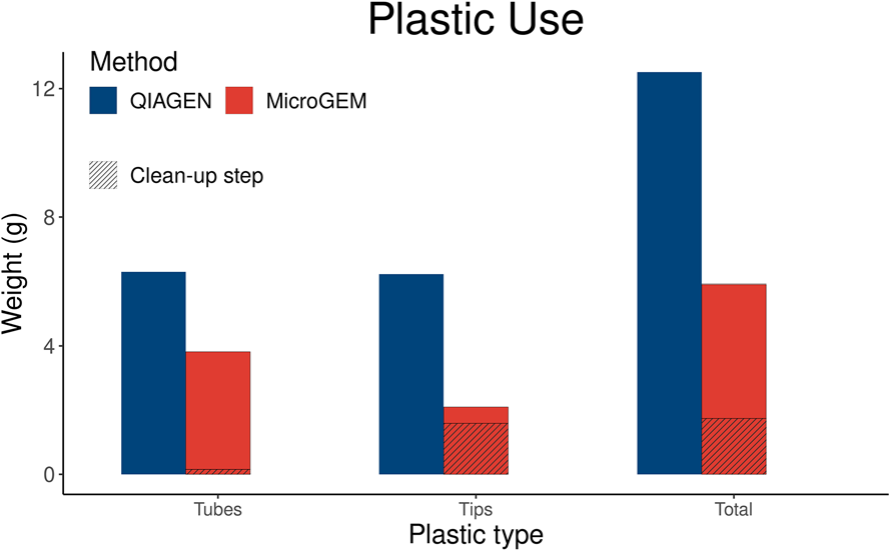
Total plastic required to process one sample by QIAGEN (blue) and MicroGEM (red). The clean-up step plastic use (MicroGEM only) hatched

The plastic consumed was determined for all steps used in each method, including the clean-up step using AMPureXP beads needed after the DNA isolation using MicroGEM (Fig. 3). To extract one sample, 12.51 g of plastic was used with QIAGEN and 5.92 g with MicroGEM + AMPureXP beads from both tube and tip items (Fig. 3). Overall, MicroGEM required 52.64% less plastic than QIAGEN to isolate DNA, per sample. The plastic footprint was determined for both CO_2_ emissions and energy consumption to produce the required plastic to process a single sample. The data showed that the plastic footprint was at least 50% lower with MicroGEM (Table 6).

**Table 6:** Plastic footprint of the DNA isolation methods to process one sample

	Carbon emissions (Kg CO_2_)	Energy used (Mj)
QIAGEN	0.04255	1.07498
MicroGEM	0.02011	0.50809

**Fig. 4:**
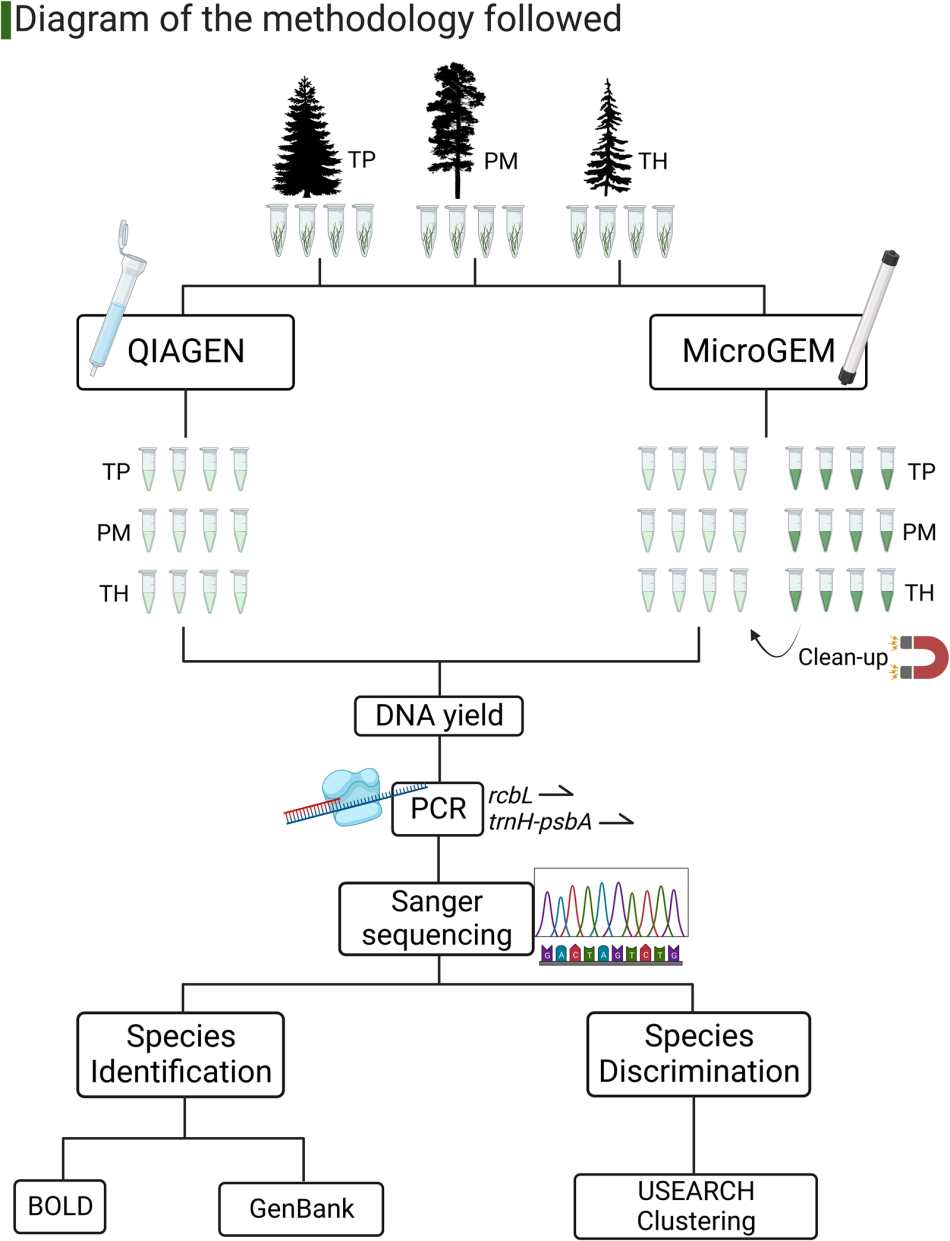
Diagram of the methodology followed. Illustration created with BioRender.com. The methodology and sequence of major steps implemented across this study, included: 1) plant material collection, 2) DNA isolation, 3) DNA yield quantification and 4) DNA quality assessment by using DNA barcoding for species identification and species discrimination by performing PCR and followed by DNA sequencing

We also measured the time required to isolate DNA with each method by determining the time needed to process 24 samples and calculated for a single sample (see methods for details). Overall, the time required to extract a single sample using MicroGEM was 51.8% less than QIAGEN (Table 7). If we only include the hands-on time needed to purify the DNA of one sample by using both methods, then MicroGEM needed 34.6% less time than QIAGEN (Fig. 4).

**Table 7:** Time required to process one sample by using each of the DNA isolation methods

	Total (min)	Total hands-on (min)
QIAGEN	119.4	5.4
MicroGEM	57.5	3.5

The plastic consumed to isolate 1146 *P. menziesii* and 79 *T. plicata* samples from the population project described above (see Table 5) by using the DNA isolation QIAGEN method was 15.3 kg, which represented 52.06 kg of CO_2_ emitted, and 1310 Mj of energy required to produce the plastic used. If MicroGEM was used to isolate the DNA of this project rather than QIAGEN, 8.1 kg less plastic would have been utilised, 27.44 kg of CO_2_ emission would have been avoided and 686 Mj of energy would have been saved. Based on an 8-h working day, 25.9 days were needed to complete the 1225 isolations using QIAGEN while only 14.7 days would have been needed to isolate the same samples if using MicroGEM.

## Discussion

The central question posed in this study was whether a rapid and plastic-efficient DNA isolation method will recover reliable DNA similarly to a commonly used kit. We compared the DNA yield, DNA quality and efficiency on four samples of three different forest tree species and considered outputs of a separate study in a larger population.

We presented data on the use of two methods to isolate nucleic acids from forest trees including method optimisations to the basic protocols to isolate the DNA compared to the original methods. No significant difference in DNA yield recovered was found between the two methods when using the improved protocols, indicating that either method will deliver sufficient DNA yield for standard molecular analyses. However, significant differences were found among species, which suggested the need for specific optimisation of laboratory protocols when looking at various species. The significant difference found in the interaction between methods and species suggests that *P. menziesii* and *T. heterophylla* recovered more DNA when using QIAGEN, but *T. plicata* recovered similar amounts in both cases. Several studies looked at the differences in amount of recovered DNA quality and time efficiency of different DNA isolation methods in plant species [37]. Bashalkhanov and Rajora [38] tested several DNA extraction systems suitable for conifers obtaining a mean DNA yield of more than 10 µg when using the QIAGEN DNeasy kit, which aligns with our DNA yield results. They also concluded that the QIAGEN DNeasy kit is not a preferred method when dealing with large numbers of samples due to the long time that is required to complete the isolation. Our initial MicroGEM DNA concentration results were similar to previous research such as described by Ryan et al. [39], which showed a very low recovered DNA concentration (less than 3 ng/µl) when using MIGROGEM on different plant tissues. After optimisation, we substantially increased the concentration to over 15 ng/µl and up to 78 ng/µl using MicroGEM. These results confirmed the utility of optimising protocols to obtain sufficient DNA to perform reliable downstream analyses. It is essential to acknowledge the relatively low number of samples in our dataset could limitate and impact our findings. However, similar sample sizes were used in comparable studies evaluating a novel DNA isolation method [40]. They found significant trends in different species with datasets of four samples per species. Nonetheless, future tests with larger sample sizes and including more species may provide further validation and enhance the generalisation of our observations.

The quality of the DNA was analysed with standard DNA Barcoding techniques with gene-specific primers as done in Armenise et al. [41] using *rbcL* and *trnH-psbA* which are commonly used to confirm the species identity and power of discrimination by PCR amplification and sequencing [42, 43]. For *trnH-psbA*, both DNA isolation methods delivered accurate size amplicons and no minor bands which match with Kress et al. [44] results, where *trnH-psbA* exhibited the highest PCR success. The sequences from *rbcL* and *trnH-psbA* markers were submitted to BOLD and GenBank, respectively, to confirm sample identities and the taxonomical assignment when using *trnH-psbA* was 100% successful, which relates to what Loera-Sánchez et al. [43] found. Those sequences of unexpected length were the only ones incorrectly identified. In the species discrimination analysis with the *trnH-psbA*, all samples from the same species clustered together, without any differences between the DNA isolation method. Several studies have validated these two loci for efficient DNA barcoding in plants [43, 44], and specifically conifers [41]. In their study, Armenise et al. [41] recovered the same expected fragment size and expected sequence length as our results when analysing *P. menziesii* using *rbcL* (710 bp) and *trnH-psbA* (565 bp) markers, showing consistency in the amplifications. Their findings suggest that combining these two markers may be preferable to perform DNA Barcoding in conifers due to their PCR uniformity and sufficient sequence quality while showing enough variation to perform species identity analyses at the genus level. In contrast, our results suggest that using *trnH-psbA* alone retrieves sufficient evidence to identify and differentiate species from different genera which corresponds with Kress et al. [44] results.

To assess the plastic and time efficiency of each DNA isolation method, we reported the plastic footprint and the time needed to process one sample. The results of this study indicate that a temperature-driven enzymatic cocktail isolation system reduces plastic footprint by 52.64% compared to a commonly used silica-based nucleic acid isolation method. This enzymatic method also reduces the average time needed to process a sample by 51.8%. When looking at the plastic footprint, we found that there is limited research on the plastic footprint that compares plastic consumption of different isolation nucleic acids methods. Marengo et al. [45] developed a DNA isolation method for plant species where they reduced the sample processing time while maintaining the quality of the DNA recovered. The authors also discussed the reduction of plastic waste achieved although this was not quantified. The lack of previous research on this matter prevents a direct comparison with our DNA isolation plastic footprint results. Nonetheless, there is an increase of studies looking at how to reduce general plastic use in laboratories [3, 19] and proposing protocols optimised to decrease the amount of plastic use [25]. Alves et al. [3] developed a 7-week study measuring the plastic used in a molecular laboratory after promoting very specific behavioural and protocol changes to reduce the use of plastic items. They were able to achieve a reduction of more than 10 kg of plastic per week on average, from an initial of 24 kg. The improved protocol for GBS library preparation proposed by Torkamaneh et al. [25] reduces both the time and plastic needed by 75% and 89%, respectively, compared to standard methods. These findings, together with our results, confirm the potential to apply sustainable measures such as using alternative materials, reusing items when possible or developing protocols which use less plastic without compromising the experiment's outcome.

Our study comparing different methods shows the potential to significantly impact on the plastic consumption and efficiency of DNA isolation in coniferous forest tree species. Our findings suggest that MicroGEM is a highly suitable method as it provided sufficient DNA yield with good quality while producing the least amount of plastic waste and being the most time-efficient. Our study also shows that selecting the most suitable method will depend on the specific requirements of the project, the species studied and the resources available. We believe that our results will encourage researchers to select DNA isolation methods based on sustainable laboratory practices, although further research is needed to explore the performance of this method on a broader range of species and molecular analyses. Additional research to assess other DNA isolation methods and their potential to reduce plastic use is also needed due to the importance of increasing the studies that evaluate the environmental impact of molecular laboratories.

## Conclusion

In conclusion, the main result of this study is that this rapid and plastic-efficient method, Plant DNA Extraction, MicroGEM, recovers sufficient and reliable DNA to perform common downstream analyses such as PCR and sequencing, and performs as well as a commonly used spin-column kit, DNeasy plant mini kit, QIAGEN. Our study highlights the merit of efforts towards developing more sustainable and efficient laboratory practices in the field of molecular biology. A reduction of plastic waste in molecular laboratories deserves further research for the development of techniques and protocols that use less single-use plastic items.

## Methods

### Plant material

The study species are the conifers *Thuja plicata* (TP), *Pseudotsuga menziesii* (PM) and *Tsuga heterophylla* (TH). They are all part of the Pinales order; *T. plicata* belongs to the Cupressaceae, whilst *P. menziesii* and *T. heterophylla* belong to the Pinaceae. Four samples per species (Fig. 4) were retrieved from different planted stands in England using an arborist slingshot following a modified method based on Youngentob et al. [46]. The leaves collected were dried by storing them in silica gel beads after removing them from the tree and until processing for DNA isolation.

### DNA isolation procedures and improvements

We isolated the DNA using 20 mg of leaf tissue from the 12 samples (Fig. 4) by using the manufacturer’s instructions for the DNeasy plant mini kit (Qiagen USA, Valencia, CA) (QIAGEN), we also modified the procedure to enhance the quality and yield of the DNA recovered, by adding 20 µl of Protease K in the lysate step and increasing the incubation time to a minimum of an hour at 55 °C. [47].

We processed the same 12 samples using the DNA isolation method Plant DNA Extraction [48] (MicroGEM) (Fig. 4) following the manufacturer’s original instructions. First, 10 mg of leaf tissue is mixed with the lysis buffer and using a mechanical homogenizer to break the cells, second, the lysate is transferred to a PDQeX DNA-binding cartridge and third, the enzymatic cocktail is added to the cartridge, which is inserted into the PDQeX device which through the combination of changes in temperature and the physical design of the cartridge performs a chemical process that releases the DNA into the recovery tubes. We also modified this procedure to optimise the DNA yield as follows: (1) The tissue was disrupted alone and added with a micro scoop to the pre-sample mix which included the lysate buffer and the enzymatic cocktail in individual tubes, (2) the sample mix was pipetted into the cartridges, which were inserted into the PDQex device with a modified plant programme with an incubation time increased from 5 to 15 min and extraction time from 5 to 10 min [49].

After recovering the DNA we performed a clean-up step by using the AMPpureXP beads [50], which use magnetic particles that bind the DNA to allow the clean-up. A concentration of 0.8 × of AMPureXP beads (Beckman Coulter™, Brea, CA, USA) was used for the size selection needed to retrieve the isolated DNA fragments (0.8 × ratio recommended by NewEngland Biolabs).

### DNA recovery

The DNA yield recovered from all samples was quantified using Qubit® Fluorometer v.4.0 (Invitrogen™, Thermo Fisher Scientific, Cleveland, OH, USA) (Fig. 1). The samples extracted by the modified protocols were used in this study (for details, see Table 2). DNA quality of these samples was visualized by electrophoresis in 0.8% Agarose gels (TAE Buffer), which confirmed the DNA presence (not shown). We performed an ANOVA test (error type iii) using the R [51] package 'ape' [52] to analyse the differences in DNA yield recovered between methods and species followed by a post-hoc Tukey’s test [53] for comparing all possible group pairings. To visualise the data, we used the R package 'ggplot2' [54].

### DNA analyses

The quality of the DNA was analysed by PCR amplification (Fig. 4) with the intergenic spacer primers situated on the chloroplast DNA: the large subunit of the ribulose-bisphosphate carboxylase gene (*rbcL*) and the plastid *trnH-psbA*, and sequencing on each sample. We adjusted the DNA concentrations to 20 ng/µl, when possible (Table 2), for PCR amplification of *rbcL* and *trnH-psbA* barcoding loci (Table 8). We used Q5® High-Fidelity 2X Master Mix DNA Polymerase (NewEngland Biolabs®, Ipswich, MA, USA) and added 100 ng of template DNA and 0.5 µM of each primer into a 25 µl final reaction following the manufacturer’s instructions. The thermocycler conditions were 98 °C for 20 s, followed by 40 cycles starting at 98 °C for 10 s, TA (*rbcL*: 54 °C and *trnH-psbA*: 63 °C, Table 8) for 30 s and 72 °C for 30 s, with a final extension at 72 °C for 2 min. The amplified regions were visualized by electrophoresis in a 1.8% Agarose gel with SYBR safe (Invitrogen™, Thermo Fisher Scientific, Cleveland, OH, USA) as stain.

**Table 8:** Locus and primer details for PCR. TA: Annealing temperature used

Marker name	Primer's sequence	TA (°C)	Expected product size (bp)	Source
*trnH-psbA*	CGCGCATGGTGGATTCACAATCC	63	500	[41]
	GTTATGCATGAACGTAATGCTC			
*rbcL*	ATGTCACCACAAACAGAAAC	54	750	[41]
	TCGCATGTACCTGCAGTAGC			

PCR products of two samples per species for each of the extraction methods were analysed by Sanger sequencing (Source Biosciences). Pre-cleaning and post-quality checks were performed by the sequencing service facilitator. Retrieved sequences were visualized and the electropherograms manually checked by using SnapGene software [55]. We exported the fasta files from the ab1 files which were trimmed with Trimmomatic [56] and aligned with MUSCLE [57].

We submitted the *rbcL* trimmed sequences to The Barcode of Life Data system v4 (BOLD) [58] and the *trnH-psbA* to the nucleotide dataset from GenBank [59]. BOLD enables a species-level identification of plant taxa by submitting queries of *MatK* and *rbcL* sequences to be searched against their reference library through their sequence threshold identification system. We verified the species discrimination efficacy of the *trnH-psbA* barcode by clustering the aligned sequences together by similarity using the software USEARCH v.11 [60]. The UCLUST algorithm divides a set of sequences into clusters providing a high-throughput species-level discrimination. The minimum similarity level was set to 99%.

### Plastic footprint and time needed for DNA isolations

The plastic used to isolate DNA with both modified methods was calculated for a single reaction by weighing every plastic item with a precision balance (Table 9) not including the packaging.

**Table 9:** Weight of plastic items required, and number of items needed per DNA isolation method to process one sample

Plastic item	Weight (g)	QIAGEN (N)	MicroGEM (N)
Cartridge	1.183	0.00	1.00
2 ml tube	1.089	1.00	1.00
1.5 mL tube	0.940	1.00	0.00
2 mL no-Lid tube	0.932	3.00	0.00
Column	0.734	2.00	0.00
0.5 mL tube	0.461	0.00	1.00
0.2 mL tube	0.153	0.00	1.00
1000 μl tip	1.033	5.13	0.17
200 μl tip	0.315	2.04	0.08
20 μl tip	0.219	0.04	0.00
10 μl tip	0.275	1.00	0.00
cut tip	0.311	0.00	1.00
Total		15.21	5.25

*N* number of items

The carbon dioxide emitted to produce 1 kg of polypropylene plastic commonly used to make laboratory supplies has been discussed by Harding et al. [61] and 3.4 kg CO_2_ is generally accepted amount. The total energy required to produce 1 kg of plastic from the extraction of raw materials to the final manufactured product is 85.9 MJ [61]. Following these data, we calculated the kg of CO_2_ emissions and total energy required to process one sample with each DNA isolation method based on the amount of plastic needed in each case.

The time required to isolate DNA by using both modified methods was timed for each step in the procedures when processing 24 samples and calculated for a single reaction. Incubation time and centrifuge steps time are fixed and remain the same for either 1 or 24 samples. We divided the hands-on time by 24 samples and added it to the measured fixed time.

## Data Availability

The datasets used and/or analysed during the current study are available from the corresponding author on reasonable request.
